# Clinical impact of an antibiotic stewardship program in a neonatal intensive care unit at a tertiary care hospital: a prospective quasi-experimental clinical study

**DOI:** 10.1186/s40780-024-00404-3

**Published:** 2025-09-23

**Authors:** Neveen Hassan Abdelaal, Nafisa Hassan Rifaat Abdel Aziz, Asmaa Mohamed Abdelaziz, Sahar Badr Hassan Khalil, Mohamed Mahmoud Mohamed Abdel-Latif

**Affiliations:** 1 Clinical Pharmacy Unit, Department of Pharmacy and Medical Supplies, Assiut University Children’s Hospital, Assiut, Egypt; 2 Department of Pediatrics, Faculty of Medicine, Assiut University, Assiut, Egypt; 3 Microbiology Unit, Department of Clinical Pathology, Faculty of Medicine, Assiut University, Assiut, Egypt; 4 Department of Clinical Pharmacy, Faculty of Pharmacy, Assiut University, Assiut, Egypt

**Keywords:** Antibiotics, Antimicrobial resistance, Antibiotic stewardship program, Neonatal intensive care unit, Neonatal sepsis.

## Abstract

**Background:**

Antimicrobial resistance represents a great global concern and initiating an antimicrobial stewardship program is one of the main efforts to control antimicrobial resistance through optimizing antimicrobial utilization. Antibiotics are used extensively and inappropriately in neonatal intensive care units (NICUs). The present study aimed to assess the clinical impact of the antibiotics stewardship program (ASP) in the NICU.

**Methods:**

The study was conducted in two phases at the NICU, Assiut University Children’s Hospital, where 1200 patients were enrolled from January 2019 to June 2020. The pre-ASP phase included making NICU-specific antibiograms, choosing the antibiotic use evaluation measures, conducting antibiotic use evaluations, and designing the ASP. The ASP intervention phase included the implementation of ASP, which involved modifying the neonatal sepsis treatment protocol according to the local antibiotic susceptibility patterns and measuring its clinical outcomes. Categorical data were tested with Pearson’s chi-squared test and Fisher’s exact test. Numerical data were tested with the Wilcoxon rank sum test.

**Results:**

A total of 603 and 597 patients were enrolled in the pre-ASP and intervention-ASP phases, respectively. The ASP intervention phase showed a significant increase in the number of C-reactive protein tests [1(1–2)vs 2(1–3),*p* = 0.001], microbiological cultures per patient [0(0–1) vs. 1(0–1),*p* = 0.001], the number of patients taking definitive therapy [15.1% vs. 20.1%,*p* = 0.023], and the number of pharmacist interventions per patient [1(1–1) vs. 1(1–2),*p* = 0.001]. The duration of the antibiotic course [9(5–16) vs. 8(4–15),*p* = 0.04] and the number of patients taking empirical therapy [38.6% vs. 30.7%,*p* = 0.004] were significantly decreased in the ASP intervention phase. The prescribing rates of antibiotics and their consumption in terms of length of therapy were changed according to the NICU-specific antibiogram; however, days of therapy were unchanged in the ASP intervention phase. There was a significant reduction in the 14-day [46% vs. 28%,*p* = 0.03] and 28-day [64% vs. 43%,*p* = 0.022] mortality of patients with late-onset sepsis after modifying the neonatal sepsis treatment protocol according to the local antibiotic susceptibility patterns in the ASP intervention phase.

**Conclusion:**

ASP implementation was successful in improving antibiotic prescribing and modifying the neonatal sepsis treatment protocol according to the local antibiotic susceptibility patterns, which resulted in reduced 14- and 28-day mortality.

**Trial registration:**

Clinical Impact of an Antibiotic Stewardship Program in a Neonatal Intensive Care Unit, Registration number NCT04039152. Registered 31 July 2019 - Retrospectively registered. https://classic.clinicaltrials.gov/ct2/show/NCT04039152.

## Introduction

Antimicrobial resistance (AMR) was stated by the World Health Organization (WHO) as one of the top 10 global public health threats facing humanity due to the continuous emergence and spread of drug-resistant pathogens that are unresponsive to the available treatments [1]. The slow development of new antimicrobials will cause AMR, resulting in great losses in human lives and national economies of all countries, as it can affect health care progress, food production, and life expectancy [2, 3].

The emergence of organisms resistant to the currently available antimicrobials is unavoidable, but the extent of propagation of the resistant organisms is governed by human behavior [4, 5]. Therefore, there is an urgent need to reduce the use of inappropriate antimicrobials to slow down the constant rise in antimicrobial resistance and preserve the utility of these life-saving drugs.

In this context, many efforts, measures, and action plans were taken worldwide to prevent the heading of a post-antibiotic era, in which common infections and minor injuries can kill once again [6]. The WHO initiated a global action plan as a global effort to control the spread of AMR. One of the main pillars of this global plan is to initiate and develop antimicrobial stewardship programs because antimicrobial misuse is recognized as the main factor that accelerates the emergence of AMR [7]. These programs are a series of coordinated interventions designed to promote the rational use of antimicrobials by improving antimicrobial prescribing practice and promoting the appropriate selection of the optimal drug regimen [8, 9]. The development and implementation of these programs in intensive care units are of great importance where antibiotics are usually the most prescribed medications, especially in neonatal intensive care units (NICUs), because neonatal sepsis is an important cause of mortality and morbidity in this age group worldwide [10, 11]. This necessitates the development of a rational approach for antibiotic use in these units. The present study aimed to assess the clinical impact of the antibiotics stewardship program (ASP) in the NICU.

## Methods

### Study design and setting

This prospective, quasi-experimental clinical study was carried out at a 48-incubator NICU of Assiut University Children’s Hospital, a 600-bed tertiary-care hospital in Egypt.

### Study patients and duration

Patients admitted to the NICU between January 2019 and June 2020 who received antibiotics for more than 72 h were included in the study. Patients whose hospital stay was less than 72 h or who did not receive any antibiotics during their hospital stay were excluded from the study. The study was conducted in two phases: the pre-ASP phase (January 2019 – December 2019) and the ASP intervention phase (January 2020 – June 2020).

### Data collection

Data was collected from patients’ medical records. Patient demographics included sex, postnatal age, gestational age according to the American Academy of Pediatrics (AAP) classification, and weight according to the WHO classification [12, 13]. The main diagnosis, comorbid conditions, presence of invasive devices (e.g., umbilical catheter, central venous catheter, urine catheter, endotracheal tube), and infection-related data (sepsis type, exposure to septic shock, site of infection) were included. Data from laboratory reports, microbiological reports, length of hospital stay, mortality at 14 and 28 days, and antibiotic therapy data (initiation date/time, route, dose, duration of therapy, and pharmacist interventions were also collected.

### Study phases

#### Pre-antimicrobial stewardship program phase

The pre-ASP phase focused on identifying and understanding antibiotic use problems through data collection, analysis, and evaluation to prepare for the ASP. In this phase, antibiotic prescribing was based on the international guidelines without the use of local NICU-specific antibiograms, thus antibiotic change was based on physicians’ preferences. There was no formal review for antibiotic prescriptions. The pre-ASP phase included:

#### Making NICU-specific antibiogram

An antibiogram was created following WHO and National Committee for Clinical Laboratory Standards guidelines [14, 15]. It was developed by collecting and analyzing NICU patients’ microbiological data from January to December 2019, by including microbial isolates found more than 30 times per year and excluding those found 30 times or less due to insufficient sample size. It analyzed antibiotic susceptibility reports to identify common pathogens and their resistance patterns, aiming to guide empiric antibiotic therapy selection based on specific resistance patterns, thus ensuring more effective treatment.

#### Choosing the measures for antibiotic use evaluation

Measures were selected to evaluate antibiotic use, based on recommendations from the IDSA, WHO, and Management Sciences for Health Organization [16–18]. The chosen metrics included:

#### Prescribing process measures

These measures involved the number of C-reactive protein (CRP) tests, microbiological cultures, antibiotic indications (prophylactic, empirical, and definitive therapy), appropriate antifungal prophylaxis (considered appropriate when administered to neonates at high risk for fungal infections, such as those with very low birth weight (< 1500 g), prolonged use of broad-spectrum antibiotics, or the presence of invasive devices), inappropriate antibiotic use (antibiotic prescriptions in which first-line treatment do not align with established clinical guidelines), number of antibiotics used per patient, antibiotic course duration, antibiotic therapy changed in 72 h (antibiotics reviewed 72 h after its administration to reassess appropriateness), antibiotic-related pharmacists’ interventions (number per patient, types, acceptance by physicians), and antibiotics prescribing rates [19–21].

#### Consumption process measures

These measures included length of therapy (total days on antibiotics, regardless of antibiotics number), days of therapy (total days on antibiotics, regardless of their doses), days of monotherapy, and antibiotic-free days per patient (total days where no antibiotics received) [19, 22, 23].

#### Clinical outcome measures

These measures involved mortality at 14 and 28 days (primary outcomes), length of hospital stay, and re-admission within 30 days of discharge (secondary outcomes) for neonatal sepsis patients. Neonatal sepsis patients were categorized by the onset of sepsis into patients with early-onset sepsis (within the first 72 h after birth), late-onset sepsis (72 h or more after birth), and hospital-acquired sepsis (within 48–72 h after hospital admission, up to 6 weeks later) [24–29].

#### Conducting antibiotic use evaluation

A cross-sectional assessment was done to check if antibiotics were used appropriately, following CDC, WHO, and Management Sciences for Health guidelines [16, 30]. The identified antibiotic use problems included inappropriate treatment protocols, inconsistent dosing, and insufficient testing (low numbers of microbiological cultures and CRP tests) among patients with suspected or proven infection. Many patients at risk of fungal sepsis were not receiving antifungal agents as a preventive measure.

#### Designing the ASP

The ASP was designed through discussions and meetings with NICU medical staff, microbiology, and clinical pharmacy departments. Key procedures for ASP implementation included:

- Modifying the neonatal sepsis treatment protocol based on the local antibiotic susceptibility patterns. The modification of treatment protocol was necessary due to the high resistance of local pathogens to commonly used antibiotics, leading to a shift to alternative antibiotics as first-line treatment based on the local antibiogram.
- Arrangements with the microbiology lab to ensure 24-hour availability for microbiological cultures and testing only the susceptibility of antibiotics that are available and suitable for neonates.
- Educating the NICU healthcare team about optimal antibiotic use steps and actions of ASP, and the modified neonatal sepsis treatment protocol.
- Training sessions for NICU nurses on proper sampling techniques.

### The antimicrobial stewardship program intervention phase

The ASP intervention phase aimed to solve the problems identified in the pre-ASP phase by implementing targeted interventions and measuring their impact. The ASP intervention phase involved:

#### Implementation of the ASP

The ASP was implemented following IDSA, SHEA, and CDC guidelines [17, 31]. The implementation was based on an enablement intervention strategy due to the ease of its integration into the normal workflow, and it was well accepted by the healthcare team. A multidisciplinary team of NICU staff conducted regular reviews of antibiotic prescriptions and provided daily feedback to the physicians in the clinical rounds to promote adherence to the ASP guidelines.

The ASP interventions included:

- Modifying the neonatal sepsis treatment protocol, especially for late-onset sepsis, to match the local antibiotic susceptibility patterns. This was guided by WHO and AAP guidelines [14, 28, 32, 33].
- Standardizing antibiotic dosing protocols.
- Educative sessions were held for NICU staff to enhance their understanding of ASP principles, steps, actions, and the importance of appropriate antibiotic use. NICU nurses were also trained in proper sampling techniques.

#### Measuring ASP outcomes

The impact of the ASP was measured by comparing the process and clinical outcome measures of the pre-ASP and ASP intervention phases. The evaluations of ASP interventions were based on the neonatal sepsis treatment protocol developed from the WHO and AAP treatment guidelines in the pre-ASP phase and according to the modified treatment protocol in the ASP intervention phase.

### Data analysis

Data were analyzed by R software statistical programming language (version 4.0.3, Vienna, Austria). Data normality was tested with the Shapiro‒Wilk normality test, which showed that the data were not normally distributed. Categorical data were expressed as numbers and percentages and were tested with Pearson’s chi-squared test and Fisher’s exact test. Noncontinuous data were described as medians and interquartile ranges and were compared with the Wilcoxon rank sum test with continuity correction for numerical data. A *P* value < 0.05 was considered statistically significant.

Only the sepsis type with a significant mortality difference between ASP phases was further analyzed to understand its predictors and outcomes. A Univariate analysis assessed the significance of all infection-related predictors for mortality outcomes, using Pearson’s chi-squared test and Fisher’s exact test. Significant predictors from the univariate analysis were included in a multivariate logistic regression to identify key predictors of improved survival. To avoid overfitting, the multivariate analysis was limited to 11 factors, chosen based on their statistical significance in the univariate analysis and clinical relevance to neonatal sepsis outcomes. The variables were considered significant predictors if the *P* value < 0.05. The association of significant variables with mortality was reported as odds ratios (OR) with a 95% confidence interval (CI) for crude odds ratios and adjusted odds ratios.

## Results

### Demographic characteristics of the study patients

A total of 1644 patients were admitted to the NICU during the study period, of whom 1200 patients were enrolled in the study, including 603 patients enrolled in the pre-ASP phase and 597 patients in the ASP intervention phase. Out of 1200 enrolled patients, 627 patients were diagnosed with neonatal sepsis (324 patients in the pre-ASP phase versus 303 patients in the intervention-ASP phase). Sepsis patients were 326 early-onset sepsis (EOS), 272 late-onset sepsis (LOS), and 29 hospital-acquired sepsis (HAS). Males were more prevalent among neonatal sepsis patients in both phases, where 202 (62.5%) out of 324 patients in the pre-ASP phase versus 190 (62.7%) out of 303 patients in the ASP intervention phase were males. The total study patients, including patients with neonatal sepsis in both phases, showed insignificant differences in their demographic characteristics (Table 1). Additionally, there was an insignificant difference in the number of EOSs, LOSs, and HASs in both phases. The intervention-ASP phase showed a significant increase in the number of culture-proven bacterial sepsis in (*P =* 0.003). Regarding, the primary site of infection most neonatal sepsis patients in both phases had a non-specific primary site of infection (Table 2).

**Table 1:** Demographic characteristics of the study patients

Patients demographic	All Study Patients (*n* = 1200)			Patients with neonatal sepsis (*n* = 627)		
	Pre-ASP phase (*n* = 603) *N* (%)	ASP intervention phase (*n* = 597) *N* (%)	*P* value	Pre-ASP phase (*n* = 324) *N* (%)	ASP intervention phase (*n* = 303) *N* (%)	*P* value
**Gender** Ambiguous Female Male	1 (0.2) 224 (37.1) 378 (62.7)	2 (0.3) 223 (37.4) 372 (62.3)	0.946 ^b^	1 (0.3) 121 (37.3) 202 (62.3)	1 (0.3) 112 (37) 190 (62.7)	0.994 ^b^
**PNA (days)** Median (IQR)	1 (1–6)	1 (1–6)	0.409 ^c^	1 (1–8)	2 (1–10)	0.189 ^c^
**GA category** Late preterm (34–36 weeks) Preterm (≤ 33 weeks) Term (≥ 37 weeks)	137 (22.7) 195 (32.3) 271 (44.9)	139 (23.3) 203 (34) 255 (42.7)	0.729 ^a^	68 (21) 130 (40.1) 126 (38.9)	65 (21.5) 119 (39.3) 119 (39.3)	0.975 ^a^
**Weight Category** ELBW (< 1 kg) LBW (< 2.5 kg) NBW (> 2.5 kg) VLBW (< 1.5 kg)	55 (9.1) 223 (37) 221 (36.7) 104 (17.2)	44 (7.4) 216 (36.2) 229 (38.4) 108 (18.1)	0.677 ^a^	45 (13.9) 126 (38.9) 90 (27.8) 63 (19.4)	25 (8.3) 111 (36.6) 99 (32.7) 68 (22.4)	0.086 ^a^
IUGR	24 (4)	23 (3.9)	1 ^a^	15 (4.6)	15 (5)	0.999 ^a^

^a^ Chi-square test, ^b^ Fisher’s exact test, ^c^ Wilcoxon rank sum test, postnatal age (PNA), gestational age (GA), normal birth weight (NBW), low birth weight (LBW), very low birth weight (VLBW), extremely low birth weight (ELBW), intrauterine growth retardation (IUGR), IQR (Interquartile range)

**Table 2:** Sepsis characteristics and presentation in neonatal sepsis patients (*n* = 627)

Sepsis characteristics	Pre-ASP phase (*n* = 324) *N* (%)	ASP intervention phase (*n* = 303) *N* (%)	*P* value
**Onset**			
Early-onset	168 (51.9)	158 (52.1)	1 ^a^
Late-onset	138 (42.6)	134 (44.2)	0.74 ^a^
Hospital-acquired	18 (5.6)	11 (3.6)	0.339 ^a^
**Presentation**			
Culture-proven	91 (28.1)	120 (39.6)	**0.003** ^a^
Septic shock	112 (34.6)	109 (36)	0.776 ^a^
**Primary site of infection**			
Lung	42 (13)	59 (19.5)	**0.031** ^b^
Gastrointestinal	35 (10.8)	19 (6.3)	
Skin	5 (1.5)	4 (1.3)	
Renal	3 (0.9)	2 (0.7)	
Central nervous system	2 (0.6)	7 (2.3)	
Blood	1 (0.3)	2 (0.7)	
Bone	3 (0.9)	**-**	
Non- specific	233 (71.9)	210 (69.3)	

^a^Chi-square test, ^b^Fisher’s exact test

### The pre-ASP phase

The study was conducted in two phases the pre-ASP phase and the intervention ASP phase. The pre-ASP phase focused on identifying and understanding antibiotic use problems to prepare for the ASP.

Making a NICU-specific antibiogram was the first step in the pre-ASP phase. It displays the in-vitro susceptibility of isolated bacterial strains from the lab data to various antibiotics. The study involved 218 bacterial isolates from neonatal sepsis patients, with 124 (56.9%) being Gram-negative and 94 (43.1%) Gram-positive. The Gram-negative isolates were Klebsiella Pneumoniae (73 isolates), Acinetobacter baumannii (22 isolates), Escherichia coli (14 isolates), Pseudomonas aeruginosa (9 isolates), Enterobacter (2 isolates), Burkholderia species (1 isolate), Proteus (2 isolates) and Salmonella (1 isolate). The Gram-positive isolates were Coagulase-negative staphylococcus (48 isolates), Staphylococcus aureus (39 isolates), Streptococcus pneumoniae (4 isolates), Staphylococcus epidermidis (2 isolates), and Enterococcus (1 isolate). The antibiogram included 160 out of the 218 bacterial isolates being reported more than 30 times per year, excluding less frequently reported isolates. The pathogens included were Klebsiella pneumoniae 73 (45.6%) isolates, Coagulase-negative staphylococcus 48 (30%) isolates, and Staphylococcus aureus 39 (24.4%) isolates. The antibiotic susceptibility patterns toward the microbial isolates of patients with neonatal sepsis are shown in Table 3.

**Table 3:** Antibiotics susceptibility pattern toward the microbial isolates of patients with neonatal sepsis (*N* = 160)

Tested antibiotics	Klebsiella Pneumoniae (*N* = 73) Susceptibility (%)	Coagulase-negative staphylococci (*N* = 48) susceptibility (%)	Staphylococcus aureus (*N* = 39) susceptibility (%)
Penicillin	0	13.3	0
Ampicillin	0	13.2	0
Ampicillin/ Sulbactam	2.4	12.5	9.1
Amoxicillin	0	-	-
Amoxicillin/ Clavulanate	3.8	0	0
Cephalexin	0	0	-
Cefuroxime	0	-	-
Cefadroxil	0	0	-
Cefaclor	0	0	-
Cefoxitin	12.7	27.6	0
Cefoperazone	0	-	-
Ceftriaxone	12.7	16.2	3.1
Cefotaxime	7.7	12.5	0
Ceftazidime	9.1	5.3	0
Cefepime	11.8	24	20.8
Aztreonam	15.4	0	0
Meropenem	30	60	0
Imipenem/cilastatin	22.2	0	-
Ertapenem	14.3	20.8	5.6
Piperacillin/tazobactam	17.3	43.2	21.2
TMP/SMX	0	0	100
Ciprofloxacin	32	45.9	39.4
Gentamycin	33.9	50	66.7
Amikacin	36.4	-	-
Vancomycin	-	71.4	66.7
Linezolid	-	91.4	86.2
Clindamycin	-	64.5	71.9
Azithromycin	-	21.7	25
Erythromycin	-	12	30
Teicoplanin	-	29.7	28.1
Rifampin	50	66.7	100
Streptomycin	50	71.4	71.4

TMP/SMX (Trimethoprim/sulfamethoxazole), *N* (number of isolates), - (susceptibility testing not performed), 0 (no susceptibility shown upon testing)

**Fig. 1:**
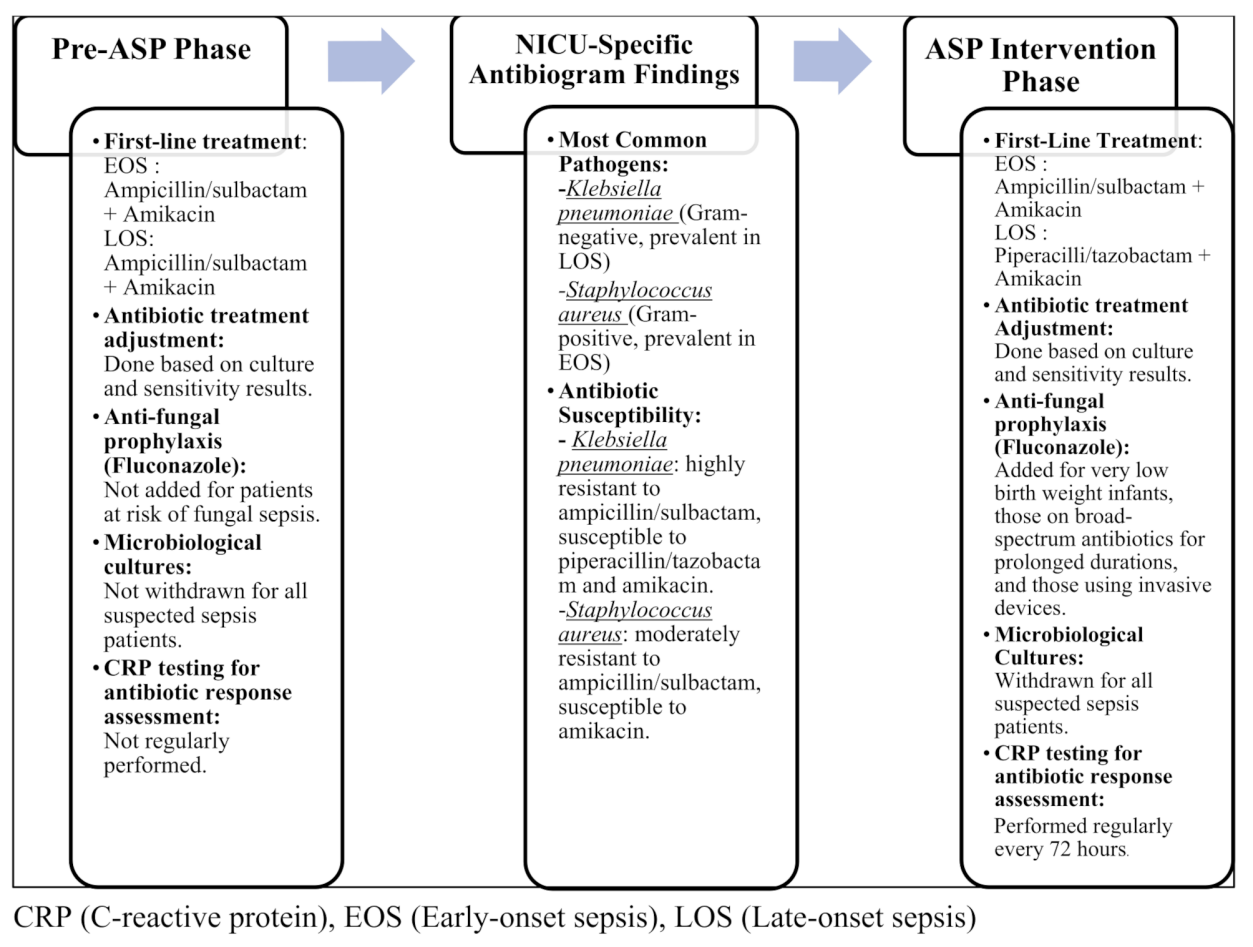
The differences in the neonatal sepsis treatment protocol in the pre-ASP phase and ASP intervention phase based on the findings of the NICU-specific antibiogram

The ASP was designed in the pre-ASP phase through discussions and meetings with NICU medical staff, microbiology, and clinical pharmacy departments to introduce the implementation procedures of ASP, agreeing on an enablement intervention strategy to be the basis of ASP implementation. The prospective audit and feedback by NICU pharmacists and physicians were agreed to be done during the daily clinical rounds. The meetings discussed the results of the constructed NICU-specific antibiogram and identified the best potential interventions that can be applied for antibiotic use optimization. The NICU medical staff approved several steps for ASP implementation, including:

- Modifying the neonatal sepsis treatment protocol especially for late-onset sepsis, to match the local antibiotic susceptibility patterns. The susceptibility patterns revealed that Klebsiella pneumoniae was the most common Gram-negative pathogen, especially in patients with late-onset sepsis (Table 4), it was highly resistant to ampicillin/sulbactam but susceptible to piperacillin/tazobactam and amikacin (Table 3). Coagulase-negative staphylococcus was the most prevalent Gram-positive pathogen in our antibiogram data, but it is less clinically significant compared to Staphylococcus aureus and is often considered a contaminant, especially in blood cultures, due to its presence on the skin. Thus, Staphylococcus aureus was prioritized and considered the most clinically significant Gram-positive pathogen in our setting due to its higher pathogenic potential and its association with severe infections, particularly since both pathogens had equal prevalence in patients with early-onset sepsis (Table 4). Staphylococcus aureus was moderately resistant to ampicillin/sulbactam and susceptible to amikacin (Table 3). This data led to the modification of the neonatal sepsis treatment protocol where the first-line antibiotics for late-onset sepsis patients were changed according to the susceptibility profiles of the most prevalent pathogen among these patients. The protocol included the introduction of piperacillin/tazobactam for empirical treatment of late-onset sepsis instead of ampicillin/sulbactam with amikacin based on the antibiogram data while continuing to use them for early-onset sepsis as recommended by the guidelines of WHO and AAP. The protocol also included adding antifungal agents as prophylaxis for patients at risk of fungal sepsis, withdrawing microbiological cultures for all patients suspected to have sepsis, and regular assessment of antibiotic response by CRP testing. The differences in the neonatal sepsis treatment protocol before and after the introduction of ASP interventions and the rationale behind the protocol changes are shown in Fig. 1.
- Implementing a new antibiotic dosing protocol.
- Arrangements with the microbiology lab ensured 24/7 sample collection for cultures, to avoid any delay in culture withdrawal. The ASP design also involved creating a list by the pharmacy unit of all the available antibiotics for neonatal infections, approved by the NICU medical staff, and provided to the microbiological lab for susceptibility testing on NICU patient isolates.
- Education of the health care team about the optimal antibiotics use, steps, and actions of implementing ASP, the modified neonatal sepsis treatment protocol was agreed to be provided once monthly then through case-based education during the daily rounds of the health care team. Training sessions were introduced for the NICU nurses focused on correct blood culture sampling procedures to avoid contamination. The schedule and content for health care team training during the ASP intervention phase are illustrated in Table 5.

**Table 4:** The causative microbial isolates of culture-proven neonatal sepsis

Causative organism	Total microbial isolates *N* = 234 *N* (%)	EOS *N* = 103 *N* (%)	LOS *N* = 117 *N* (%)	HAS *N* = 14 *N* (%)	*P* value
Klebsiella pneumoniae (G-ve)	66 (28.2)	23 (22.3)	36 (30.8)	7 (50)	0.25
Acinetobacter baumannii (G-ve)	24 (10.3)	11 (10.7)	12 (10.3)	1 (7.14)	0.71
Escherichia coli (G-ve)	9 (3.8)	5 (4.9)	4 (3.4)	-	0.89
Enterobacter (G-ve)	3 (1.3)	-	3 (2.6)	-	0.84
Pseudomonas aeruginosa (G-ve)	4 (1.7)	2 (1.9)	2 (1.7)	-	0.79
S. maltophilia (G-ve)	4 (1.7)	2 (1.9)	2 (1.7)	-	0.99
CoNS (G + ve)	62 (26.5)	27 (26.2)	31 (26.5)	4 (28.6)	0.75
S. aureus (G + ve)	51 (21.8)	27 (26.2)	23 (19.7)	1 (7.14)	0.85
Enterococcus (G + ve)	4 (1.7)	3 (2.9)	1 (0.8)	-	0.6
Staph. epidermidis (G + ve)	2 (0.9)	1 (1)	1(0.8)	-	0.99
Strept. pneumoniae (G + ve)	4 (1.7)	1 (1)	2 (1.7)	1 (7.14)	0.66
Aerococcus viridans (G + ve)	1 (0.4)	1 (1)	-	-	0.94
**Total Gram-positive** **bacteria**	124 (53)	60 (58.3)	58 (49.6)	6 (42.9)	0.46
**Total Gram-negative** **bacteria**	110 (47)	43 (41.7)	59 (50.4)	8 (57.1)	0.39
**Fungi**	11 (4.7)	3 (2.9)	8 (6.8)	-	0.86

CoNS (Coagulase-negative staphylococci), EOS (Early-onset sepsis), G + ve (Gram-positive bacteria), G -ve (Gram-negative bacteria), HAS (Hospital-acquired sepsis), LOS (Late-onset sepsis), N = number of isolates, S. aureus (Staphylococcus aureus), Staph. epidermidis (Staphylococcus epidermidis), S. maltophilia (Stenotrophomonas maltophilia), Strept. pneumoniae (Streptococcus pneumoniae)

### Antibiotic prescribing process measures

During the study, 627 (52.2%) patients of the 1200 enrolled patients received antibiotics for treating clinical or culture-proven neonatal sepsis, while 573 (47.8%) received antibiotics as prophylaxis against sepsis. The assessment of antibiotic use was performed by evaluating antibiotic prescribing and antibiotic consumption process measures. The assessment of antibiotics prescribing showed improvements in the ASP intervention phase, with a significant increase in the number of microbiological cultures per patient [0 (0–1) versus 1 (0–1), *P* = 0.001], the percentage of definitive antibiotics therapy (15.1% vs. 20.1%, *P* = 0.023) and appropriate antifungal prophylaxis (12% vs. 28.4%, *P* = 0.004) in the ASP intervention phase. There was a significant decrease in empiric antibiotic therapy (38.6% vs. 30.7%, *P* = 0.004), and inappropriate antibiotic use (42.6% vs. 0%, *P* = 0.00004) in the ASP intervention phase. The ASP intervention phase also showed a significant increase in the number of CRP tests per patient [1 (1–2) versus 2 (1–3), *P* = 0.001] and a significant decrease in antibiotic course duration [9 (5–16) vs. 8 (4–15), *P* = 0.04]. However, there was no significant difference in the change of antibiotic therapy within 72 h or the number of antibiotics used per patient (Table 6).

The ASP intervention phase showed the involvement and contribution of the pharmacists in the ASP. There was a significant increase in the number of pharmacist interventions per patient [1 (1–1) vs. 1 (1–2), *P* = 0.001], the number of dose optimizations (88.2% vs. 96.8%, *P* = 0.0003), and a significant decrease in renal dosing adjustments (16.6% vs. 7.8%, *P =* 0.0033) in the ASP intervention (Table 7).

Assessment of the antibiotic prescribing rates showed a significant increase in the prescribing rates of amikacin (71.3% vs. 91.8%, *P* = 0.001) and piperacillin/tazobactam (0.8% vs. 41.4%, *P* = 0.001) in the ASP intervention phase. The prescribing rates of ampicillin/sulbactam (89.2% vs. 58.8%, *P* = 0.001), vancomycin (32.7% vs. 22.1%, *P* = 0.001), cefotaxime (64.8% vs. 53.8%, *P* = 0.001), ceftazidime (5.3% vs. 1.8%, *P* = 0.002) and cefepime (18.1% vs. 7.4%, *P* = 0.001) were significantly decreased in the ASP intervention phase (Table 8).

**Table 5:** Antibiotic prescribing process measures in the pre-ASP and ASP intervention phases

Prescribing process measures	Pre-ASP phase	ASP intervention phase	*P* value
**Antibiotics indications, ***N* (%)	*N* = 603	*N* = 597	
Prophylaxis Empiric Definitive	279 (46.3) 233 (38.6) 91 (15.1)	294 (49.5) 183(30.7) 120 (20.1)	0.302 ^a^ **0.004** ^a^ **0.023** ^a^
Number of CRP tests/patient, Median (IQR)	1 (1–2)	2 (1–3)	**0.001** ^c^
Number of microbiological cultures/patients, Median (IQR)	0 (0–1)	1 (0–1)	**0.001** ^c^
**Antibiotic administration**	*N* = 603	*N* = 597	
Antibiotic therapy changed in 72 h, N (%)	247 (41)	247 (41.4)	0.931 ^a^
Number of antibiotics used per patient, median (IQR)	3 (2–4)	3 (2–4)	0.172 ^c^
Antibiotic course duration (days), Median (IQR)	9 (5–16)	8 (4–15)	**0.04** ^c^
**Neonatal sepsis patients**	*N* = 324	*N* = 303	
Appropriate antifungal prophylaxis N (%)	39 (12)	86 (28.4)	**0.004** ^c^
Inappropriate antibiotic use N (%)	138 (42.6)	0	**0.00004** ^b^

^a^ Chi-square test, ^b^ Fisher’s exact test, ^c^ Wilcoxon rank-sum test, CRP (C-reactive protein), IQR (interquartile range)

**Table 6:** Pharmacist interventions in the pre-ASP and ASP intervention phases

Pharmacist interventions	Pre-ASP Phase *N* = 229	ASP intervention phase *N* = 282	*P* value
Number of interventions/patients, Median (IQR)	1 (1–1)	1 (1–2)	**0.001** ^b^
Accepted interventions, *N* (%)	222 (96.9)	276 (97.9)	0.703 ^a^
Dose optimization interventions, *N* (%)	202 (88.2)	273 (96.8)	**0.0003** ^a^
Renal dosing adjustments interventions, *N* (%)	38 (16.6)	22 (7.8)	**0.0033** ^a^

*N* = number of pharmacist interventions, ^a^ Chi-square test, ^b^ Wilcoxon rank-sum test, IQR (interquartile range)

**Table 7:** Antimicrobial prescribing rates for each prescribed antimicrobial in the pre-ASP and ASP intervention phases

Prescribed antimicrobials	Pre-ASP phase *N* = 603 *N* (%)	ASP intervention phase *N* = 597 *N* (%)	*P* value
Amikacin	430 (71.3)	548 (91.8)	**0.001** ^a^
Amoxicillin/clavulanate	20 (3.3)	22 (3.7)	0.849 ^a^
Ampicillin/sulbactam	538 (89.2)	351 (58.8)	**0.001** ^a^
Piperacillin/tazobactam	5 (0.8)	247 (41.4)	**0.001** ^a^
Vancomycin	197 (32.7)	132 (22.1)	**0.001** ^a^
Linezolid	21 (3.5)	20 (3.4)	1 ^a^
Cefotaxime	391 (64.8)	321 (53.8)	**0.001** ^a^
Ceftazidime	32 (5.3)	11 (1.8)	**0.002** ^a^
Cefoperazone	23 (3.8)	34 (5.7)	0.163 ^a^
Ceftriaxone	3 (0.5)	1 (0.2)	0.624 ^b^
Cefepime	109 (18.1)	44 (7.4)	**0.001** ^a^
Meropenem	69 (11.4)	52 (8.7)	0.14 ^a^
Imipenem/cilastatin	0	4 (0.7)	0.06 ^b^
Azithromycin	1 (0.2)	1 (0.2)	1 ^b^
Clarithromycin	1 (0.2)	3 (0.5)	0.371 ^b^
Clindamycin	1 (0.2)	0	1 ^b^
Metronidazole	120 (19.9)	118 (19.8)	1 ^a^
Fluconazole	44 (7.3)	100 (16.8)	**0.001** ^a^

^a^ Chi-square test, ^b^ Fisher’s exact test

### Antibiotic consumption process measures

Antibiotic consumption was assessed in terms of length of therapy (LOT) in days for each antibiotic used per admission. The LOTs of ampicillin/sulbactam [6 (4–10) vs. 5 (3–9), *P* = 0.001], vancomycin [10 (4–15) vs. 8.5 (3.75-14), *P* = 0.001], and cefotaxime [6 (3–12) vs. 6 (3–11), *P* = 0.0007] significantly decreased in the ASP intervention phase. The LOTs of amikacin [4 (2–5) vs. 4 (3–5), *P* = 0.001], piperacillin/tazobactam [7 (3–8) vs. 8 (5–12), *P* = 0.001], ceftazidime [5.5 (3-9.25) vs. 8 (6.5–12), *P* = 0.001] and cefepime [8 (3–13) vs. 10 (3.75-18), *P* = 0.001] significantly increased in the ASP intervention phase. There was also a significant increase in days of monotherapy in the ASP intervention phase [3 (1–8) vs. 5 (2–8), *P* = 0.0003] (*P* = 0.0003). However, the days of therapy of the antibiotics used during the patient’s hospital stay and antibiotic-free days showed no significant difference between the pre-ASP and the ASP intervention phases (Table 9).

**Table 8:** Quantitative antimicrobial consumption for each prescribed antimicrobial in terms of length of therapy (days)

Antimicrobials	Pre-ASP phase *N* = 603 Median (IQR)	ASP intervention phase *N* = 597 Median (IQR)	*P* value
Amikacin	4 (2–5)	4 (3–5)	**0.001**
Amoxicillin/clavulanate	6 (3–8)	8 (6–16)	0.690
Ampicillin/sulbactam	6 (4–10)	5 (3–9)	**0.001**
Piperacillin/tazobactam	7 (3–8)	8 (5–12)	**0.001**
Vancomycin	10 (4–15)	9 (4–14)	**0.001**
Linezolid	9 (7–13)	9 (2–17)	0.889
Cefotaxime	6 (3–12)	6 (3–11)	**0.0007**
Ceftazidime	6 (3–9)	8 (7–12)	**0.001**
Cefoperazone	6 (3–10)	8 (5–12)	0.117
Ceftriaxone	9 (5–12)	3 (3–3)	0.321
Cefepime	8 (3–13)	10 (4–18)	**0.001**
Meropenem	8 (3–14)	10 (5–14)	0.139
Imipenem/cilastatin	-	10 (5–15)	0.044
Azithromycin	2 (2–2)	5 (5–5)	0.994
Clarithromycin	5 (5–5)	5 (5–6)	0.311
Clindamycin	20 (20–20)	-	0.320
Metronidazole	9 (4–14)	9 (5–17)	0.997
Fluconazole	8 (3–13)	5 (2–10)	**0.001**
Days of therapy	17 (9–32)	16 (8–30)	0.120
Antibiotic-free days	2 (1–7)	2 (1–6)	0.438
Days of Monotherapy	3 (1–8)	5 (2–8)	**0.0003**

Chi-square test, IQR (interquartile range)

### Clinical outcome measures

The assessment of clinical outcome measures for patients with LOS showed a significant decrease in mortality at 14 days (46% vs. 28%, *p* = 0.03) and 28 days (64% vs. 43%, *p* = 0.022), but there was no significant difference in the length of hospital stay or 30-day re-admission between the pre-ASP and intervention-ASP phases. However, for EOS and HAS patients, there was no significant difference in 14- and 28-day mortality, length of hospital stays, or 30-day re-admission between the pre-ASP phase and the ASP intervention phases (Table 10).

**Table 9:** Clinical outcomes among neonatal sepsis patients in the pre-ASP and ASP intervention phases

Clinical outcome measures	Pre-ASP phase *N* = 324	ASP intervention phase *N* = 303	*P* value
**Early-onset sepsis**	*N* = 168	*N* = 158	
14-day mortality, *N* (%)	96 (57.1)	78 (49.4)	0.19^a^
28-day mortality, *N* (%)	107 (63.7)	95 (60.1)	0.58^a^
Length of hospital stay (days), Median (IQR)	9 (5–19)	9 (4–16)	0.75^c^
30-day Readmission, *N* (%)	-	1 (0.6)	0.48^b^
**Late-onset sepsis**	*N* = 138	*N* = 134	
14-day mortality, *N* (%)	46 (33.3)	28 (20.9)	**0.03** ^a^
28-day mortality, *N* (%)	64 (46.4)	43 (32.1)	**0.02** ^a^
Length of hospital stay (days), Median (IQR)	13 (7–24)	12 (7–23)	0.58^c^
30-day Readmission, *N* (%)	3 (2.2)	4 (3)	0.72^b^
**Hospital-acquired sepsis**	*N* = 18	*N* = 11	
14-day mortality, *N* (%)	2(11.1)	-	0.51^b^
28-day mortality, *N* (%)	7 (38.9)	3 (27.3)	0.69^b^
Length of hospital stay (days), Median (IQR)	24 (16–32)	30 (22–40)	0.23^c^
30-day Readmission, *N* (%)	-	-	-

^a^Chi-square test, ^b^Fisher’s exact test, ^c^Wilcoxon rank-sum test, IQR (interquartile range)

**Table 10:** Univariate model assessing the significance of all infection-related predictors for mortality outcomes

Infection risk factors categories	Variable (risk factor) Name	Variable total number	Variable among dead patients *N* (%)	X^2^	df	*P*- Value	OR	CI
**Neonatal**	**Low birth weight**	438	272 (62.1%)	20.963	1	0.0005	2.233	1.578–3.159
	**Prematurity**	382	231 (60.5%)	7.447	1	0.006	1.568	1.134–2.167
**Obstetric**	**Cesarean delivery**	478	271 (56.7%)	0.251	1	0.616	1.099	0.759–1.591
	**Multiple births**	136	79 (58%)	0.268	1	0.605	1.107	0.754–1.625
	**Bad obstetrics** **history**	71	37 (52.1%)	0.527	1	0.468	0.833	0.508–1.366
	**Premature rupture of membranes**	119	66 (55.5%)	0.027	1	0.868	0.967	0.647–1.445
	**Chorioamnionitis**	3	2 (66.7%)	0.136	1	0.999	1.566	0.141–17.357
	**Antenatal antibiotic** **exposure**	14	9 (64.3%)	0.386	1	0.534	1.417	0.469–4.277
**Medical History**	**Previous hospitalization**	218	123 (56.4%)	0.11	1	0.917	1.018	0.731–1.417
	**Surgery exposure**	125	72 (57.6%)	0.135	1	0.713	1.077	0.725–1.6
**Invasive Devices**	**Umbilical catheter**	304	202 (66.5%)	25.460	1	0.0005	2.284	1.653–3.156
	**Central venous** **catheter**	34	26 (76.5%)	6.034	1	0.014	2.662	1.186–5.976
	**Urine catheter**	52	32 (61.5%)	0.671	1	0.413	1.275	0.712–2.283
	**Feeding tube**	33	18 (54.5%)	0.036	1	0.850	0.934	0.462–1.889
	**Endotracheal tube**	370	294 (79.5%)	199.352	1	0.0005	13.273	9.021–19.529
**Infection Origins**	**Blood**	3	3 (100%)	2.355	1	0.26	0.559	0.522–0.6
	**Bone**	3	3 (100%)	2.355	1	0.26	0.559	0.522–0.6
	**Central nervous** **system**	9	6 (66.7%)	0.211	1	0.646	1.572	0.39–6.344
	**Gastrointestinal**	54	25 (46.3%)	2.325	1	0.127	0.649	0.370–1.135
	**Lung**	101	41 (40.6%)	11.817	1	0.001	0.472	0.306–0.729
	**Skin**	9	4 (44.4%)	0.507	1	0.515	0.621	0.165–2.334
	**Renal**	5	2 (40%)	0.533	1	0.658	0.518	0.086–3.122
	**Non- specific**	443	268 (60.5%)	11.634	1	0.001	1.823	1.289–2.579
**Infection severity**	**Culture-proven sepsis**	211	101 (47.9%)	8.840	1	0.003	0.604	0.432–0.843
	**Gram-negative** **infections**	109	42 (38.5%)	16.613	1	0.0005	0.421	0.275–0.643
	**Gram-positive** **infections**	102	59 (57.8%)	0.143	1	0.705	1.086	0.707–1.669
	**Septic shock**	221	198 (89.6%)	155.113	1	0.0005	14.087	8.751–22.675
	**Infection-induced renal dysfunction**	20	16 (80%)	4.776	1	0.029	3.226	1.066–9.763
	**Infection-induced hepatic dysfunction**	27	14 (51.9%)	1.572	1	0.39	0.835	0.386–1.806
**Antimicrobials used in** **treatment**	**Ampicillin/sulbactam**	474	278 (58.7%)	4.968	1	0.026	1.514	1.050–2.183
	**Piperacillin/tazobactam**	153	74 (48.4%)	4.968	1	0.026	0.66	0.458–0.952
	**Antifungal prophylaxis**	125	90 (72%)	15.949	1	0.0005	2.356	1.535–3.613
**Drug Resistance patterns**	**MDR**	144	62 (43.1%)	12.998	1	0.0005	0.503	0.345–0.733
	**Drug-sensitive**	23	6 (26.1%)	8.758	1	0.003	0.263	0.102–0.677
	**XDR**	44	33 (75%)	6.836	1	0.009	2.483	1.231–5.008
**Pathogen Resistance patterns**	**CRE**	15	8 (53.3%)	0.049	1	0.825	0.890	0.319–2.486
	**ESBL**	5	4 (80%)	1.165	1	0.392	3.149	0.350–28.339
	**MRCONS**	25	10 (40%)	2.755	1	0.097	0.507	0.224 − 1.146

**Multiple births**: pregnancy with more than one baby at a time. **Bad obstetric history**: problems in previous pregnancies, including miscarriage, stillbirth, or any other unwanted conditions. **Premature rupture of membranes**: when your water breaks more than 18 h before labor starts. **Chorioamnionitis**: infection of the fetal membranes and amniotic fluid. **Antenatal Antibiotic Exposure**: Fetal exposure to antibiotics during pregnancy. **Non-specific infections**: infections where the causative pathogen is not identified. **Feeding tubes**: refer to feeding tubes that involve surgical procedures to place the tube directly into the stomach or intestines, such as gastrostomy, jejunostomy, nasojejunal, or gastrojejunostomy. **Culture-proven sepsis**: Sepsis diagnosed with microbiological cultures confirming the presence of infection, rather than clinical signs alone. **Antifungal prophylaxis**: refers to using antifungal agents to prevent fungal sepsis. **MDR (Multi-drug resistance)**: refers to organisms resistant to multiple antibiotics, but still susceptible to at least one antibiotic in three or more categories. **XDR (Extensive-drug resistance)**: refers to organisms resistant to almost all antibiotics, but still susceptible to at least one antibiotic in two or fewer categories. **Drug-sensitive**: refers to organisms that can be inhibited or killed by antibiotics, being resistant to at least one agent in one or two categories). **Pathogen Resistance pattern**: was identified as reported in the microbiological cultures as CRE (carbapenem-resistant Enterobacteriaceae), ESBL (extended-spectrum beta-lactamase), and MRCoNS (methicillin-resistant coagulase-negative staphylococci)

Late-onset sepsis was the only type showing a significant difference in 14- and 28-day mortality between ASP phases, prompting a detailed analysis of its mortality predictors and outcomes. Out of 38 predictors assessed via univariate analysis, 17 were statistically significant, including low birth weight, prematurity, use of umbilical catheters, central venous catheters and endotracheal tubes, the occurrence of infection-induced renal dysfunction, septic shock, lung infections, non-specific infections, culture-proven sepsis, Gram-positive infections, use of ampicillin/sulbactam and piperacillin/tazobactam as first-line treatment, use of antifungal prophylaxis, presence of multi-drug resistance, extensive-drug resistance, and drug-sensitive patterns Table 11. A multivariate logistic regression was then conducted to ascertain the effects of these significant predictors on mortality, incorporating 11 of these 17 predictors to avoid overfitting based on the degree of their statistical significance and clinical relevance to mortality outcomes. The 11 predictors included in the regression model were the use of central venous catheters and endotracheal tubes, the occurrence of infection-induced renal dysfunction, septic shock, lung infections, use of ampicillin/sulbactam and piperacillin/tazobactam as first-line treatment, use of antifungal prophylaxis, presence of multi-drug resistance, extensive-drug resistance, and drug-sensitive patterns. Six out of the 11 predictors included remained significant in the multivariate model including endotracheal tube use, occurrence of septic shock, infection-induced renal dysfunction, lung infections, use of piperacillin/tazobactam as first-line treatment, and presence of multi-drug resistance pattern. The odds ratios of mortality significantly decreased by 34% with using piperacillin/tazobactam as first-line treatment (OR = 0.66, 95% CI 0.458–0.952), insignificantly increased by 27% with using ampicillin/sulbactam as first-line treatment (OR = 1.27, 95% CI 0.764–2.111), and insignificantly decreased by 30% with using antifungal prophylaxis for patients at risk of developing fungal sepsis (OR = 0.7, 95% CI 0.390–1.264). The effects of other predictors on the odds ratios of mortality are shown in Table 12.

**Table 11:** Multivariate regression model identifying the key predictors of improved survival among late-onset sepsis patients (*N* = 272)

Variable Name	B	S.E.	Chi (X^2^)	df	*P* value	Odds Ratio	95% CI
Endotracheal tube	2.229	0.244	83.331	1	0.0005	9.292	5.758–14.995
Central venous catheter	0.973	0.517	3.536	1	0.060	2.646	0.960–7.296
Infection-induced renal dysfunction	1.650	0.685	5.796	1	0.016	5.205	1.359–19.934
Septic shock	1.932	0.275	49.518	1	0.0005	6.903	4.030- 11.823
Lung infections	− 1.297	0.306	17.898	1	0.0005	0.273	0.150–0.499
Piperacillin/tazobactam	− 0.415	0.187	4.936	1	0.026	0.660	0.458–0.952
Ampicillin/sulbactam	0.239	0.259	0.848	1	0.357	1.270	0.764–2.111
Antifungal prophylaxis	− 0.354	0.300	1.391	1	0.238	0.702	0.390–1.264
MDR	-0.521	0.260	4.021	1	0.045	0.594	0.357–0.988
XDR	0.482	0.444	1.180	1	0.277	1.620	0.679–3.866
Drug-sensitive	-0.832	0.670	1.543	1	0.214	0.435	0.117–1.617

**Lung infections**: refer to infections of lung origin. **Antifungal prophylaxis**: refers to using antifungal agents for prophylaxis from fungal sepsis. **MDR (Multi-drug resistance)**: Organisms resistant to multiple antibiotics (at least remain susceptible to one in three or more categories). **XDR (Extensive-drug resistance)**: Organisms resistant to almost all antibiotics (at least remain susceptible to one in two or fewer categories). **Drug-sensitive**: organisms that can be inhibited or killed by antibiotics (resistant to at least one agent in one or two categories)

**Table 12:** Training schedule and content for health care team during the ASP intervention phase

Profession	Training Topic	Educational Content	Frequency
**Physicians**,** Pharmacists** **and Nurses**	Steps and Actions of ASP	- Detailed explanation of the ASP protocols, including guidelines for antibiotic prescribing, monitoring, and de-escalation strategies.	Initial comprehensive training followed by monthly refresher sessions.
**Physicians and Pharmacists**	Optimal Antibiotics Use	- Education on selecting the most appropriate antibiotics based on infection type, patient history, and local antibiogram data.	Monthly workshops then case-based discussions during daily rounds.
	Modified neonatal sepsis treatment protocol	- Training on updated treatment protocols for neonatal sepsis, including first-line and second-line antibiotic choices, and criteria for switching antibiotics based on culture results. - Training on the use of CRP testing to monitor the effectiveness of antibiotic therapy and guide treatment decisions.	Monthly training sessions then case-based discussions during daily rounds.
**Nurses**	Proper sampling techniques	Instructions on correct methods for collecting and handling samples to ensure accurate microbiological testing.	Weekly hands-on workshops.

CRP (C-reactive protein)

## Discussion

Antimicrobial stewardship program implementation in the NICU is crucial for controlling AMR. The study assessed antibiotic utilization, identified antibiotic use problems, and implemented ASP interventions to manage these problems. The findings indicated that ASP had a direct positive impact on clinical outcomes for neonatal sepsis patients in the NICU.

In the present study, 627 out of 1200 patients were diagnosed with sepsis. Among these patients, 326 (52%) had EOS, 272 (43.4%) had LOS, and 29 (4.6%) had HAS. Neonatal sepsis incidence rates vary globally due to differences in socioeconomic levels, healthcare facilities, infection control protocols, diagnostic methods, and antibiotic use. The incidence rates in our study were higher than those reported in Brazil, Indonesia, India, Pakistan, Nepal, Ethiopia, and other Egyptian studies [34–42]. However, the incidence of EOS in this study was lower than in Nepal and India but higher than in previous Egyptian studies [34–36, 39, 43–45]. The study also showed a lower incidence of LOS and HAS compared to India and other Egyptian studies [34–36, 45, 46]. In contrast, developed countries reported very low rates of neonatal sepsis due to high-quality healthcare services [36, 47]. Males constituted 62.5% of neonatal sepsis patients in the present study, supporting the hypothesis that males may be more susceptible to infection due to sex steroid differences that influence susceptibility to infection [36, 48–52]. Identifying that male infants were more susceptible to sepsis may help tailor the ASP to address specific vulnerabilities whenever possible. Understanding the epidemiology of neonatal sepsis incidence, prevalence, and patterns helped in recognizing the disease burden and the need for an effective ASP. Highlighting these epidemiological differences allowed for evaluating healthcare practices’ impact on infection rates and enabled targeted interventions within the ASP. The epidemiological data supported informed decision-making, resource allocation, and policy development, allowing optimal treatment and improved clinical outcomes for neonatal sepsis patients. This information was crucial for assessing the ASP’s effectiveness in enhancing neonatal care in the NICU.

The NICU-specific antibiogram developed in our study included the identification of bacterial pathogens and their antibiotic susceptibility profiles. The antibiogram data were crucial in guiding empiric antibiotic therapy and evaluating their appropriateness based on the susceptibility patterns, ensuring effective treatment against the identified pathogens. The antibiogram’s key impacts included a more precise selection of empiric antibiotics upon which the neonatal sepsis treatment protocol was modified, thus it helped in reducing the incidence of inappropriate antibiotic use and improving the clinical outcomes. It also served as a valuable tool for the ASP team during regular review and feedback sessions, facilitating evidence-based decision-making and promoting adherence to ASP guidelines. The study highlights the importance of using local antibiotic susceptibility data which was crucial for the ASP to develop relevant and effective guidelines and interventions. Overall, the study encourages tailoring antibiotic therapy to local susceptibility data to ensure effective antibiotic use, and improved patient care, and to our knowledge, no other studies highlighted the impact of using local susceptibility data in empiric antibiotic therapy.

The study assessed antibiotic prescribing and usage among 1200 patients, finding that 52.2% received antibiotics for sepsis treatment and 47.8% for sepsis prophylaxis. It highlighted significant antibiotic exposure due to common practices of sepsis prevention and combined therapies, with higher rates compared to other studies [53, 54]. High antibiotic exposure is typically linked to overall high consumption or frequent use [55]. Understanding antibiotic use patterns and the reason behind high exposure is essential for optimizing antibiotic use, effective ASP implementation, and accurate assessment of ASP outcomes.

The prescribing process assessment in our study found that after implementing the ASP, there was a significant increase in microbiological cultures per patient, appropriate antifungal prophylaxis, and definitive antibiotic therapy, while empiric therapy and inappropriate antibiotic use significantly decreased, showing prescribers’ adherence to ASP guidelines. Prophylactic antibiotic use did not change significantly in the ASP intervention phase. There was also a significant increase in CRP tests per patient and a significant decrease in antibiotic course duration, indicating better antibiotic response follow-up and antibiotic use optimization efforts. The success of ASPs relies on multidisciplinary collaboration and prescribers’ attitudes, which are crucial for optimal antibiotic use and better neonatal sepsis management [56, 57].

The ASP intervention phase showed a significant increase in pharmacist interventions per patient and dose optimization interventions, highlighting their growing role in improving antibiotic use. Renal dosing adjustments for vancomycin decreased significantly due to a new dosing protocol based on the patient’s serum creatinine instead of age. The high acceptance of pharmacists’ interventions indicated effective collaboration with physicians, though this collaboration remained consistent across ASP phases. Previous studies emphasize pharmacists’ crucial role in optimizing prescribing behavior and monitoring antimicrobial use, with pharmacist-led ASPs showing improved antibiotic use in NICUs [58–60]. Therefore, effective collaboration between pharmacists and prescribers positively influences antibiotic use and ASP outcomes [61].

The current study showed significant changes in the prescribing rates and consumption of commonly used antibiotics in the NICU, aligning with local antibiotic susceptibility data and ASP recommendations. The ASP also included fluconazole for fungal sepsis prevention in at-risk patients. During the ASP intervention phase, fluconazole consumption decreased significantly despite an increase in its prescribing rates, due to prophylactic dosing twice weekly. Many studies have described differences in antibiotic susceptibility patterns across countries [62–64]. The present study uniquely assessed changes in antibiotic use based on local data, there was no change in overall antibiotic consumption, but a significant increase in monotherapy days during the ASP intervention phase, as the interventions did not focus on stopping antibiotics for neonates without sepsis signs or symptoms.

Many studies have shown that ASPs effectively reduce antibiotic use in NICUs [54, 65–67]. A global study emphasized the need for worldwide implementation of NICU-specific ASPs due to their success in lowering antibiotic use [54]. Another study demonstrated that ASPs in NICUs safely reduced antibiotic exposure by ensuring proper treatment and minimizing unnecessary use [65]. In Greece, a 3-year study across 15 public NICUs found that ASP interventions reduced antibiotic use, particularly by stopping antibiotics for neonates without sepsis signs or symptoms [66]. Additionally, a study reported a decrease in antibiotic use and the proportion of neonates receiving antibiotics from the third to the sixth day of life by implementing a 48-hour automatic stop order for those without sepsis signs [67]. Another study demonstrated that ASPs in hospitalized neonates effectively reduced antibiotic consumption by limiting broad-spectrum antibiotics and shortening treatment durations [68]. This context highlights the importance of ASP interventions in NICUs and changes in antibiotic use based on local susceptibility data. This approach can also lead to more cost-effective treatments by avoiding unnecessary or ineffective antibiotics and help in combating antibiotic resistance by minimizing the use of broad-spectrum antibiotics.

The study found no significant difference in 14- and 28-day mortality for EOS and HAS patients between the pre-ASP and ASP intervention phases. However, for LOS, there was a significant decrease in 14- and 28-day mortality during the ASP intervention phase, attributed to using piperacillin/tazobactam as empiric antibiotic therapy based on local antibiotic susceptibility data. Hospital stay length and 30-day re-admission rates showed no significant difference for EOS, LOS, and HAS between ASP phases. A retrospective cohort study found no association between ASP and changes in antibiotic duration or clinical outcomes in NICU [69]. Another study on pediatric patients, including neonates, showed ASP recommendations were linked to shorter hospital stays but not higher mortality or re-admission [70]. No other studies have evaluated the impact of ASP implementation on neonatal sepsis mortality.

The present study also examined the associations between changing the treatment protocol for late-onset sepsis based on local antibiotic resistance patterns and the improved clinical outcomes of these patients. It found that the key mortality predictors included endotracheal tube use, occurrence of septic shock, infection-induced renal dysfunction, lung infections, use of piperacillin/tazobactam as first-line treatment, and presence of multi-drug resistance pattern. Piperacillin/tazobactam reduced mortality by 34%, making it potentially more effective than ampicillin/sulbactam. Even a modest increase in susceptibility rates (17.3% for piperacillin/tazobactam versus 2.4% for ampicillin/sulbactam) can improve the clinical outcomes. Additionally, the broader antimicrobial spectrum of piperacillin/tazobactam, combined with its efficacy in treating co-infections and preventing secondary infections is likely contributed to its clinical success in reducing mortality of critically ill neonates. Furthermore, the modification of the neonatal sepsis treatment protocol by introducing ASP intervention, employing piperacillin/tazobactam as the first-line treatment and incorporating antifungal prophylaxis were associated with better clinical outcomes. These findings highlight the importance of tailored antimicrobial strategies and the adjustment of treatment protocols based on local antibiotic resistance patterns to optimize clinical outcomes in NICUs. Various studies have shown that factors linked to neonatal sepsis mortality differ significantly among NICUs due to varying maternal, neonatal, and healthcare-associated risk factors [71–73]. It is crucial to eliminate, reduce, and manage these factors appropriately to treat neonatal sepsis effectively and improve its clinical outcomes. However, no other studies have explored the impact of such protocol changes on neonatal sepsis mortality.

The study emphasizes the positive impact of ASP on clinical outcomes while acknowledging certain limitations. The limitations included the inability to assess AMR patterns and the potential confounding of ASP outcome measures due to the relatively short study duration. The number of HAS patients was very low to an unbiased estimate. Use of CRP testing for antibiotic response evaluation instead of procalcitonin testing, which was unavailable. The study did not specify sepsis-attributable mortality, only reporting the proportion of neonatal sepsis patients who died without specifying the underlying causes. Additionally, the study was single-centered, which limits generalizability to other NICUs.

## Conclusion

The implementation of the ASP was feasible and associated with improved antibiotic prescribing and patient outcomes. The pre-ASP phase was successful in identifying the antibiotic use problems as well as the resistance pattern of the commonly used antibiotics in the NICU. The ASP intervention phase showed a change in the antibiotic prescribing rates and their consumption levels according to the local antibiotic susceptibility pattern.

Additionally, modifying the neonatal sepsis treatment protocol in the ASP intervention phase resulted in reduced 14- and 28-day mortality in patients with LOS. This study may help medical staff implement ASP effectively in NICUs and encourage them to adopt their clinical practice according to their local antibiotic susceptibility patterns for better patient outcomes.

## Data Availability

All data generated and/or analyzed during the current study are included in this article. The datasets used and/or analyzed during the current study are available from the corresponding author upon reasonable request.

## Abbreviations

AAP: American Academy of Pediatrics
AMR: Antimicrobial resistance
ASP: Antibiotics Stewardship program
CDC: Centers for Disease Control and Prevention
CRP: C-reactive protein
CoNS: Coagulase-negative staphylococcus
EOS: Early-onset sepsis
HAS: Hospital-acquired sepsis
IDSA: Infectious Diseases Society of America
LOS: Late-onset sepsis
LOT: Length of therapy
MDR: Multi-drug resistance
NICU: Neonatal Intensive Care unit
SHEA: The Society for Healthcare Epidemiology of America
WHO: World Health Organization
XDR: Extensive drug resistance
